# Psychometric evaluation of the Chinese version of the febrile convulsion knowledge scale for parents/caregivers: translation and validation study

**DOI:** 10.1186/s12912-024-02073-x

**Published:** 2024-06-17

**Authors:** Yuxiu Liu, Lan Zhang, Xiaotong Yan, Xin Wang, Yuqi Huang

**Affiliations:** 1 School of Nursing, Jinzhou Medical University, Jinzhou, China; 2 Department of Nursing, First Affiliated Hospital of Jinzhou Medical University, Liaoning, Jinzhou, China; 3 Department of Nursing, Huaian Hospital of Huaian City, 223200 Huaian, Jiangsu, China

**Keywords:** Caregiver, Febrile convulsion, Factor analysis, Psychometric properties background

## Abstract

**Background:**

Fever is one of the most common clinical symptoms of respiratory diseases in children. Once the child has a fever, parents and caregivers are mainly concerned that the child may have a febrile convulsion. A lack of cognitive ability not only leads to anxiety but also aggravates or delays the time of children’s medical treatment and even seriously affects the prognosis because of improper management of fever patients.Therefore, it is necessary to clarify the degree of mastery of knowledge related to febrile convulsions, implement targeted guidance and health education, and ensure that parents and caregivers receive correct and reasonable first aid treatment. The purpose of this study was to translate the Febrile Convulsion Knowledge Scale for Parents/Caregivers into Chinese and to verify its reliability and validity for Chinese parents and caregivers of children.

**Methods:**

The Brislin traditional translation model was used to translate the Febrile Convulsion Knowledge Scale for Parents/Caregivers from English to Chinese, following authorization from the original author of the scale. This involved literal translation, back translation, and cultural adaptation. A convenience sampling method was used to select 402 parents and caregivers of children in the pediatric ward and pediatric infusion clinic of a Grade III hospital in Liaoning Province. The item analysis method was employed to assess item differentiation, while the Delphi method was used to analyze content validity. Scale reliability was evaluated through the calculation of internal consistency and test-retest reliability. Exploratory and confirmatory factor analyses were conducted to explore and verify the underlying factor structure and scale validity.

**Results:**

The Chinese version of the Febrile Convulsion Knowledge Scale for Parents/Caregivers consists of 3 dimensions and 8 items. The Cronbach’s alpha coefficient was 0.828, with each dimension having coefficients of 0.806, 0.720, and 0.702. The split-half reliability and test-retest reliability were 0.716 and 0.790, respectively. The Chinese version has good reliability. Exploratory factor analysis revealed that the Bartlett sphericity test was 394.52 (*p* < 0.001) and that the KMO value was 0.802 > 0.600, indicating suitability for factor analysis. Principal component analysis and orthogonal rotation of maximum variance were performed on the data, and items with a load greater than 0.40 within a single factor were selected for inclusion. The resulting three-factor structure explained 70.78% of the total variance. All model fitting indices were within the acceptable range, indicating the good structural validity of the Chinese version. The results of both exploratory and confirmatory factor analyses support this conclusion.

**Conclusions:**

The Chinese version of the Febrile Convulsion Knowledge Scale for Parents/Caregivers has good reliability and validity. It can be used as a tool for clinical pediatric nurses to evaluate the knowledge of parents and caregivers of children with febrile convulsion and provide the basis for the design and implementation of targeted training plans according to the results obtained from the Chinese scale.

Febrile convulsion (FC), also known as febrile seizures, refers to seizures caused by fever (rectal temperature ≥ 38.5 °C, axillary temperature ≥ 38 °C), without central nervous system infection or other causes of seizures, and without a previous history of febrile seizures. It is one of the most common childhood emergencies and the most common neurological disease in childhood [1, 2]. The age of first onset is mainly between 6 months and 5 years, and the incidence is greater between 12 months and 18 months [3]. The current prevalence is approximately 2-4% [4]. The main clinical symptoms are sudden loss of consciousness accompanied by local or systemic muscle tonic and clonic convulsions [5]. FC often occurs when parents and caregivers are unprepared. Particularly at the time of the first attack, they often panic when they see the child having a sudden seizure and convulsing all over the body. In addition to not understanding this first emergency, they may resort to calling the child’s name loudly, shaking the child vigorously, or putting their fingers in the child’s mouth [6]. These behaviors can cause additional injuries to children, such as bites and fractures, and delay the time for timely treatment [7–9]. When faced with a range of child manifestations, parents and caregivers may feel more stressed and worried about the potential impact on the child’s intellectual and behavioral development. This can lead to negative emotions such as anxiety, depression and irritability [10, 11] and affect the quality of life of the whole family.

Therefore, a measuring instrument is needed to evaluate the knowledge of parents and caregivers with FC so that clinical pediatric nurses can carry out targeted knowledge education for parents and caregivers.However, there is a lack of objective measurement tools in the Chinese version. Therefore, the purpose of this study was to introduce the Febrile Convulsion Knowledge Scale for Parents/Caregivers into China, use a simple and effective tool to quickly assess the knowledge of Chinese parents and caregivers with FC, identify knowledge blind areas and misunderstandings, and provide an objective basis for the development and implementation of targeted training programs for pediatric nurses.

## Methods

### Study design and participants

A cross-sectional study was conducted from November 2023 to January 2024 to evaluate the Febrile Convulsion Knowledge Scale for Parents/Caregivers. The parents and caregivers of children in a Grade III hospital in Liaoning were selected as the study objects by a convenience sampling method.The inclusion criteria were as follows: (1) aged 18 years or older; (2)parents or caregivers (Other than parents who provide free care and support to the child and are related by blood or law. Such as grandfather, grandmother, etc.) who voluntarily agreed to participate in the study; (3) cared for at least one child aged 0–18 years. Individuals with cognitive and reading disabilities were excluded. To ensure the reliability of the analysis results, according to the principle that the sample size should be 5–10 times the variable, the sample size of EFA should be at least 100 cases, and the sample size of CFA should be at least 200 cases [12]. Considering a sample loss rate of 20% [13], the final sample size was 148–296. A total of 410 questionnaires were sent out in this study, and 402 were effectively collected, resulting in an effective recovery rate of 98.04%.

### Instruments

#### General demographic characteristics questionnaire

According to the research purpose, the researchers designed a general data questionnaire, including age, relationships with children, educational level, job, income, etc.

#### Febrile convulsion knowledge scale for parents/caregivers

The scale was developed by Fatma Toksoz in 2023 [14] on the basis of the literature [15, 16], with a total of 8 items and 3 dimensions. Basic knowledge (1.2.3.4), first aid knowledge (5.6), and long-term effects (7.8). A 5-point Likert scale was used, and the correct items were scored from 1 to 5 (1 = “strongly disagree,” 2 = “disagree,” 3 = “neutral,” 4 = “agree,” and 5 = “strongly agree”). Questions(2.3.5.6.7.8) are reverse scored from 5 − 1. The total score on the scale ranges from 8 to 40, with lower scores indicating poorer knowledge of FC among family members.

### Procedures

#### Scale translation and cross-cultural adaptation procedure

In this study, Fatma Toksoz, the original author of the questionnaire, was contacted by email and authorized to translate the Febrile Convulsion Knowledge Scale for Parents/Caregivers [14]. The original scale was translated and cross-culturally adapted strictly following the Brislin translation model [17] to form the Chinese version of the Febrile Convulsion Knowledge Scale for Parents/Caregivers. The specific steps were as follows: (1) Translation: The original English scale was translated into Chinese versions (T1,T2 ) by two bilingual researchers whose mother tongue was Chinese on the research team. The research team discussed the two Chinese versions of the questionnaire, discussed and resolved the controversial points, and integrated the first draft of the Chinese version(T3). (2) Back translation: Two bilingual native English teachers who had never been exposed to the source questionnaire translated T3 into English versions (E1, E2), which were combined into English version ET3. Finally, E3 was sent to the original authors of the scale for comments, and then the Chinese version (C1) was formed after discussion and modification with members of the research team. (3) Cultural adjustment: In November 2023, the Chinese version of scale C1 was culturally adjusted through the Delphi letter consultation method. Six experts (3 clinical pediatric nursing experts and 3 nursing management experts), all of whom had associate senior professional titles and a bachelor’s degree or above, were invited to participate. The number of years worked by the experts was 24.67 ± 3.44, all of which were more than 15 years. Combined with clinical work experience and professional theory, the context, cultural adaptability, and language expression of the C1 items in the Chinese version of the scale were evaluated with reference to the original scale. Finally, the research team modified the questionnaire according to expert opinions and developed the Chinese version (C2).

#### Data collection procedure

The questionnaire is available in two forms: online and paper. Participants scan the QR code to answer the questions online via the Questionnaire Star platform. Informed consent was obtained from caregivers of children before the investigation, and an anonymous collection method and voluntary principles were adopted. The questionnaires were distributed to parents and caregivers of children who met the inclusion and exclusion criteria via a convenience sampling method. The questionnaire was completed independently by the participants. Prior to completing the questionnaire, participants were informed of the purpose and significance of the study, how to complete the questionnaire, and were instructed not to complete the questionnaire incorrectly, erroneously, or confusingly. After completing the questionnaire, the data were collected. A total of 410 parents and caregivers completed the questionnaire. Invalid questionnaires, such as those for which only one option was repeated for all questions or for which there was obvious logical confusion, were screened out, and 402 valid questionnaires were ultimately obtained, for an effective recovery rate of 98.04%. Two weeks later, 30 parents and caregivers randomly selected from the participants used the same questionnaire again to assess the retest reliability of the Chinese version [18].

### Data analysis

SPSS 25.0 and AMOS 23.0 were used for data entry and analysis. Normally distributed measurement data are represented by mean values (standard deviations, SD), and enumeration data are described by percentages. The critical ratio method and correlation coefficient method were used for item analysis. Cronbach’s alpha coefficient, split-half reliability, and test-retest reliability were used for reliability analysis. The validity test was assessed using content validity and construct validity. The content validity of the I-CVI and S-CVI. Exploratory factor analysis (EFA) and confirmatory factor analysis (CFA) were used to assess construct validity.

#### Item analysis

The critical ratio method and correlation coefficient method were used to screen the items. (1) Critical ratio method: The 402 questionnaires were sorted according to the total score from high to low, T-test was used to compare whether the difference between the high group (the top 27%) and the low group (the bottom 27%) was statistically significant [19]. When the critical ratio was > 3.000, the discriminability of the item was high [19]. (2) Correlation coefficient method: The correlation coefficient between 8 items and the translated scale was calculated. Whether each item of the translated scale could be retained was judged by the item-total score correlation (*r* > 0.4) [20] and Cronbach’s alpha coefficient of the deleted item.

#### Reliability analysis

Reliability is an indicator of the accuracy or consistency of the response of the measuring instrument to the measurement result, reflecting the true degree of the measured characteristics. That is, whether the measurement tool is true and consistent for the measured object or the measured variable. The more consistent the test results, the smaller the error and the higher the reliability [21]. Cronbach’s alpha coefficient, split-half reliability, and test-retest reliability were used to evaluate the reliability of the scale. Cronbach’s alpha coefficient of the translated scale and each dimension was calculated, and 0.70 was taken as the acceptable standard of the reliability coefficient to evaluate the internal consistency of the scale [22]. The intraclass correlation coefficient (ICC) was used to evaluate the test-retest reliability of the scale. The 30 parents of the children were retested two weeks later using the translated scale and analyzed with the results of the first measurement to assess the temporal stability and consistency of the scale. To measure the stability and consistency of the scale over time.

#### Validity analysis

Six experts were selected to score each item of the Chinese version of the Febrile Convulsion Knowledge Scale for Parents/Caregivers from the perspective of content importance. A 4-point Likert scale was used(1 to 4 corresponding to “irrelevant” to “very relevant”). The item-level content validity index (I-CVI) and scale-level content validity index (S-CVI) were calculated based on the expert rating results. The I-CVI refers to the proportion of the total number of experts who give 3 or 4 points to a project; the S-CVI is the mean of the I-CVI for all items.Generally, I-CVI ≥ 0.780 and S-CVI ≥ 0.900 are required [21].

The parents and caregivers of 402 children were randomly divided into two groups: one group (*n* = 150) for EFA and the other group (*n* = 252) for CFA. In EFA, we need to pay attention to the following points [23]: (1) Kaiser‒Meyer‒Olkin (KMO) value > 0.600; (2) the Bartlett sphericity test was significant (*p* < 0.05); (3) there was no obvious double loading of

the heading item; (4) title item load > 0.500; (5) cumulative variance interpretation > 50%. Principal component analysis (PCA) was used to rotate and delete items with factor loads < 0.500 by the varimax method to extract common factors with eigenvalues > 1 [24]. In CFA, the model fitting indices mainly include the chi-square degree of freedom(χ2/df), root-mean-square error of approximation (RMSEA), root mean square residual (RMR), comparative fit index (CFI), goodness-of-fit index (GFI), norm fit index (NFI), Tuck-Lewis index (TLI), and value added fit index (IFI). It is generally believed that χ2/df < 3.000, RMSEA < 0.080, RMR < 0.050, CFI, GFI, NFI, TLI, and IFI > 0.900 meet the model fitting requirements [25].

## Results

### Scale translation and cross-cultural adaptation

According to the actual situation and language expression habits of China, the articles in the first draft of the Chinese version are reviewed and adjusted from the perspectives of semantics, idioms, experience and concepts. Under the background of Chinese culture and language expression, the sudden loss of consciousness and convulsions caused by high fever convulsion are called spasm and convulsion by most people. In order to make it easier for parents of children with different educational levels and backgrounds to understand and avoid conceptual misunderstandings, the term “febrile convulsion” mentioned in the scale was modified to “febrile convulsion/febrile spasm”.

### Descriptive statistics

A total of 402 participants were investigated, including 240 parents (59.7%), and 162 other caregivers (40.3%). The average score of the febrile convulsion knowledge scale of Chinese parents/caregivers was 21.64 ± 4.903. Other social demographics are shown in Table 1.

**Table 1:** Frequency distribution of demographic characteristics(*n* = 402)

Factors	Group	*n*	%
**Ages(years)**	18–30	146	36.30
	31–45	132	32.80
	46–60	105	26.10
	>60	19	4.70
**The relationship with the child**	parents	240	59.70
	Other caregivers (grandparents, etc.)	162	40.30
**Education**	Primary and below	77	19.20
	Junior high school	56	13.90
	High school or technical secondary school	66	16.40
	Junior college	53	13.20
	Undergraduate course	120	29.90
	Postgraduate and above	30	7.50
**Occupation**	Be engaged in business	168	41.80
	Be a farmer	61	15.20
	Individual (business, etc.)	29	7.20
	Attend school	46	11.40
	unemployed	98	24.40
**Income situation** **RMB/month**	< 2000	147	36.60
	2000–5000	146	36.30
	5001–10000	101	25.10
	> 10000	8	2.00
**Whether the child Febrile Convulsion occur**	YES	108	26.90
	NO	294	73.10

### Item analysis

The critical ratio (CR) of the 8 items in the Chinese version of the scale ranged from 10.701 to 23.831, all > 3.000, and *P* < 0.001, indicating that the differentiation of each item was good. The score of each item was positively correlated with the total score of the scale (r) 0.560–0.760, all > 0.4 and *P* < 0.001, indicating a moderate correlation between each item and the scale. The Cronbach’s alpha coefficient of the Chinese version was 0.828. After deleting any items, the Cronbach’s alpha coefficients of the Chinese version ranged from 0.793 to 0.826, which did not exceed the total Cronbach’s alpha coefficient of the scale (Table 2). All items on the summary scale were retained.

**Table 2:** Item analysis for Chinese version of the febrile convulsion knowledge scale for parents/caregivers

Item	Item-total correlation	Critical ratio	P值	Cronbach’s Alpha if item deleted
**Knowledge1**	0.760**	23.831	<0.001	0.794
**Knowledge2**	0.764**	20.949	<0.001	0.793
**Knowledge3**	0.701**	15.083	<0.001	0.803
**Knowledge4**	0.691**	19.941	<0.001	0.809
**First-aid5**	0.590**	10.701	<0.001	0.818
**First-aid6**	0.688**	14.820	<0.001	0.805
**Long-term Effect7**	0.612**	13.815	<0.001	0.815
**Long-term Effect8**	0.560**	12.608	<0.001	0.823

**P<0.01

### Validity analysis

#### Content validity analysis

Six experts evaluated the correlation between each item on the Chinese version through a 4-point scoring method (1–4 corresponding to “irrelevant” to “very relevant”). The content validity index S-CVI of the Chinese total table was 0.958, and the content validity index I-CVI of the items ranged from 0.833 to 1.000.

#### Exploratory factor analysis

**Fig. 1:**
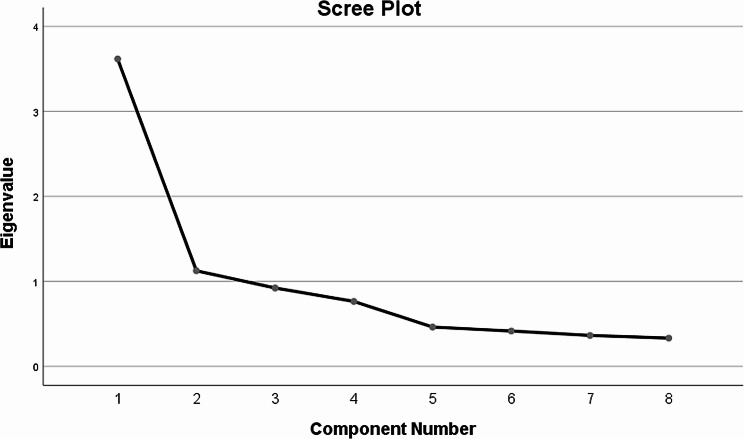
Screen plot of exploratory factor analysis for the Chinese version of the febrile convulsion knowledge scale for parents/caregivers

The KMO value was 0.802, and the Bartlett test of sphericity value was 394.52 (*p* < 0.001). This indicates that there is a good correlation between the variables, which is suitable for confirmatory factor analysis. The factor loading for each item is shown in Table 3. Three common factors with eigenvalues > 1 were extracted by orthogonal rotation of the factors by principal component analysis and the varimax method, and the loading of each item on its factor was > 0.50, so no item was removed. In addition, three factors explained 29.22%, 22.00%, and 19.56% of the variance and 70.78% of the total variance, respectively. The three principal component factors were further confirmed by a scree plot. (Fig. 1).

**Table 3:** Factor loadings of exploratory factor analysis for Chinese version of the febrile convulsion knowledge scale for parents/caregivers

Factor	Factor 1	Factor 2	Factor 3
**Knowledge1**	0.852		
**Knowledge2**	0.730		
**Knowledge3**	0.694		
**Knowledge4**	0.635		
**First-aid5**		0.891	
**First-aid6**		0.845	
**Long-term Effect7**			0.918
**Long-term Effect8**			0.694

#### Confirmatory factor analysis

**Fig. 2:**
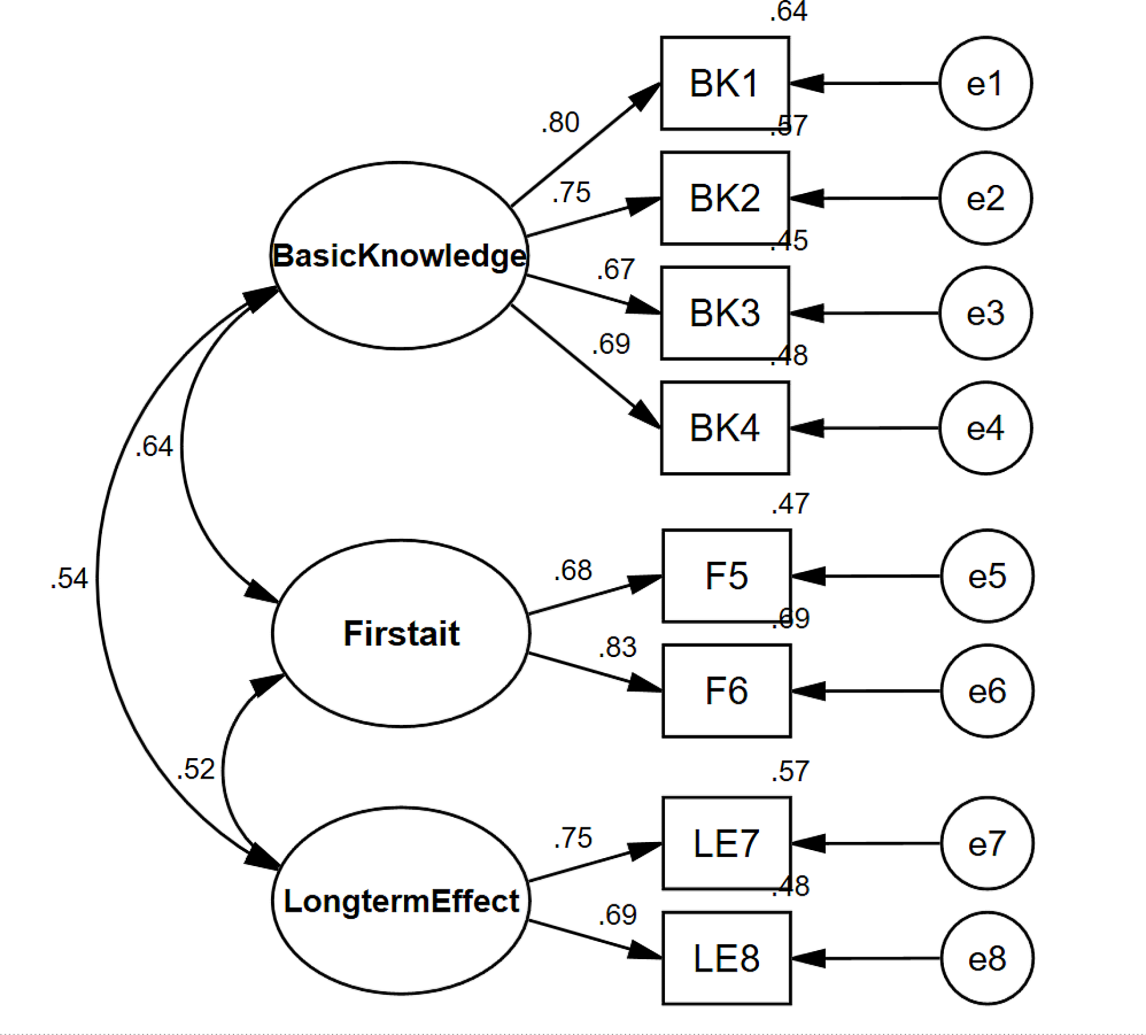
Standardized three-factor structural model of the Chinese version of the febrile convulsion knowledge scale for parents/caregivers (*N* = 252)

Amos 23.0 software was used to construct the model, and CFA was performed on the survey data to obtain the structural equation model, as shown in Fig. 2. The goodness of fit indices of the models are shown in Table 4. In conclusion, the Chinese version of the Febrile Convulsion Knowledge Scale for Parents/Caregivers has good structural validity.

**Table 4:** Fit indices of the Chinese version of the febrile convulsion knowledge scale for parents/caregivers

Fit indices	χ2/df	RMSEA	RMR	CFI	GFI	NFI	TLI	IFI
**Reference value**	<3.000	<0.080	<0.050	>0.900	>0.900	>0.900	>0.900	>0.900
**Model**	1.815	0.057	0.027	0.979	0.971	0.955	0.965	0.979

### Reliability analysis

The Cronbach’s alpha coefficient of the Chinese version was 0.828, and the values of the three dimensions were 0.806, 0.720, and 0.702. The split-half reliability was 0.716. Two weeks later, 30 parents and caregivers were randomly selected for re-evaluation, and the test-retest reliability (ICC) was 0.797 (Table 5).

**Table 5:** Reliability analysis for the Chinese version of the febrile convulsion knowledge scale for parents/caregivers

Dimension	Cronbach’s Alpha	split-half reliability	test-retest reliability
**Basic Knowledge**	0.806	0.716	0.797
**First-ait**	0.720		
**Long-term Effect**	0.702		

## Discussion

In recent years, the incidence of respiratory infectious diseases in children has increased, and children are more prone to FC due to fever. Studies have shown [10, 26] that FC in children is one of the sources of anxiety and fear for parents and caregivers. Many parents and caregivers expressed unwarranted fear of FC. The reason was that parents and caregivers have insufficient understanding of and misunderstandings about FC [27–29]. These anxieties and fears of parents and caregivers persist, but they also lead to unnecessary and inappropriate treatment, delay the time to rescue, and have a negative impact on children’s health. It is important for paediatric nurses who have regular contact with parents and caregivers to be aware of any gaps in their knowledge regarding family communication. Popularize knowledge of FC and provide guidance on how to manage the acute phase of FC in children. Compared to the “Questionnaire survey on parents’ knowledge, attitude, concern, and practice of febrile convulsions” developed by Huang et al., 45 questions were included [30]. The large number of items on the scale may pose challenges to practical administration, resulting in low participant compliance and inaccurate measurement. A more condensed version of the Febrile Convulsion Knowledge Scale for Parents/Caregivers, with the advantages of faster administration and fewer items. This scale assesses three aspects: basic knowledge, first aid knowledge, and long-term effects. It provides a simple and effective tool for clinical pediatric nurses to assess the knowledge of parents and caregivers of children with FC.

This study is the first to translate the Febrile Convulsion Knowledge Scale for Parents/Caregivers into Chinese, conduct rigorous cross-cultural debugging, and test its reliability and validity. The results showed that the Chinese version had good reliability and validity. Clinical pediatric nurses can be used to evaluate the knowledge of parents/caregivers with FC and identify deviations and misunderstandings of parents’ knowledge of FC to provide a basis for pediatric nurses to develop and implement training plans for related FC.

### The Chinese version of the scale has the suitable distinction

The Chinese version of the Febrile Convulsion Knowledge Scale for Parents/Caregivers has appropriate discrimination. Based on Brislin’s classical translation model, the Chinese version was developed through literal translation, back translation, synthesis, cross-cultural adjustment, and pre-investigation. The analysis revealed that the CR values were greater than 3.000 and *P* < 0.001, the score of each item was positively correlated with the total score of the scale, and the correlation coefficient was greater than 0.4 [31], indicating that the quality of the items in the scale was good. Moreover, after deleting each item, the Cronbach’s alpha coefficient did not exceed the original value of the translation scale, suggesting that all 8 items should be retained.

### The Chinese version of the scale has suitable reliability

In this study, three aspects of the reliability of the Chinese version of the Febrile Convulsion Knowledge Scale for Parents/Caregivers were tested: internal consistency, split-half reliability, and test-retest reliability (ICC). The results showed that the Cronbach’s alpha coefficient of the Chinese scale was 0.828, which was higher than that of the English version [14]. This may be due to differences in cultural backgrounds between countries. The importance and meticulous care given to the next generation in Chinese culture may have increased the motivation of parents and caregivers to learn about febrile seizures, thereby improving the internal consistency of the Chinese parent and caregivers groups. The Cronbach’s alpha coefficients of each dimension ranged from 0.747 to 0.790, indicating that the scale has good internal consistency and high reliability. The split-half reliability was 0.716. Two weeks later, the ICC was 0.797. An ICC > 0.75 indicated good test-retest reliability, ICC ≥ 0.4 or ≤ 0.75 indicated good test-retest reliability, and ICC < 0.4 indicated poor test-retest reliability [32]. This shows that the scale has good timing stability.

### The Chinese version of the scale has suitable validity

In this study, the validity of the scale was analyzed and evaluated in terms of content validity and construct validity. Content validity reflects whether the scale items meet the measurement purpose and requirements, while construct validity describes the matching degree between the theoretical hypothesis of the scale and the actual measurement value [33]. The I-CVI of the Chinese version was between 0.833 and 1, and the S-CVI was 0.958, which was higher than the reference value of content validity and good content validity [34]. This indicates that experts recognize the content evaluated by the scale, and the language of the scale is simple and easy to understand, which is suitable for parents and caregivers of children.

EFA and CFA were used to test the construct validity of the Chinese scale. It is generally believed that the ideal structural validity criterion (1) explains 40.00% or more of the total variation in factors extracted from exploratory analysis; (2) for each item, one factor had a high load value (> 0.400), and the other factors had low load values. The results showed that PCA and maximum variance orthogonal rotation were performed on the data, and items with individual factor loads greater than 0.40 were selected for inclusion. A total of three common factors were extracted, explaining 70.781% of the total variance. All items in the component matrix have loads above 0.5 on their respective dimensions [35]. The results of CFA showed that χ2/df = 1.606, RMSEA = 0.057, RMR = 0.027, CFI = 0.979, GFI = 0.971, NFI = 0.955, TLI = 0.965, and IFI = 0.979, and the other fitness indices were all within the acceptable range [36, 37]. The overall fit of the Chinese version of the model was good.

### Limitations

There are several drawbacks in this study that need to be considered. First, although the sample size met the research standards, only some parents and caregivers of children completed the scale, which may affect the universality and representativeness of the survey results. Second, this study selected only one area for survey research, which may not be nationally representative and is not conducive to promoting the comprehensive use of the scale. Further multicenter studies with large sample sizes are needed to explore the applicability of this scale in the future. Finally, although we have comprehensively verified the differentiation, reliability, and validity of the Chinese version among parents and caregivers. The factors that influence parents’ and caregivers’ knowledge of febrile convulsions have not been explored. Therefore, it will be of primary importance for our next work.

## Conclusions

This study strictly followed the Brislin translation model and successfully introduced the Febrile Convulsion Knowledge Scale for Parents/Caregivers, which has good reliability and validity. It also provides an effective and reliable tool for assessing the knowledge of parents and caregivers about febrile convulsions. It also lays the foundation for the development of training programs and education for pediatric nurses in the future.

## Data Availability

The datasets used and/or analysed during the current study are available from the corresponding author on reasonable request.
